# Evaluation of peak skeletal muscle perfusion in the lower extremities of athletes using arterial spin labeling

**DOI:** 10.1186/1532-429X-16-S1-P164

**Published:** 2014-01-16

**Authors:** Mitchel R Stacy, Christopher M Caracciolo, Maolin Qiu, Albert J Sinusas

**Affiliations:** 1 Internal Medicine, Yale University School of Medicine, New Haven, Connecticut, USA; 2 Diagnostic Radiology, Yale University School of Medicine, New Haven, Connecticut, USA

## Background

Arterial spin labeling (ASL) magnetic resonance imaging (MRI) allows for evaluation of skeletal muscle perfusion and provides a non-invasive index of vascular function. Previous studies have investigated ASL responses during reactive hyperemia in healthy subjects and patients with peripheral vascular disease; however, ASL has not been applied for assessment of tissue perfusion in athletes. This study evaluated regional differences in peak tissue perfusion among calf muscle groups during reactive hyperemia in college football athletes and compared ASL responses to lower body strength and functional test results.

## Methods

ASL MRI was performed on the mid-calf of 19 male athletes (19.8 ± 1.0 yrs) with a 3T magnet to evaluate peak hyperemic tissue perfusion among individual muscle groups during reactive hyperemia induced by proximal cuff occlusion. Athletes were separated by position (larger linemen vs. smaller position players) for comparison purposes. Athletes' lower body strength and jumping ability were evaluated and compared to peak skeletal muscle perfusion responses.

## Results

Linemen presented with higher body mass, body fat, and systolic blood pressure (p < 0.05). Athletes did not significantly differ in maximal lower body strength; however, position players had greater vertical and broad jump performance (p < 0.0001). In analysis of all athletes, the soleus muscle, a muscle with traditionally high capillary density, exhibited significantly higher peak perfusion during reactive hyperemia when compared to all other muscle groups (p < 0.05). Additionally, peak perfusion in the 4 calf muscles of interest was significantly greater in position players when compared to linemen and was associated with greater jumping performance.

## Conclusions

ASL MRI offers a non-invasive technique for quantifying regional differences in lower extremity tissue perfusion and may relate to vascular density as well as functional performance. Assessment of peak skeletal muscle perfusion with ASL may help to evaluate and optimize exercise training for improved lower extremity function.

## Funding

This work was supported in part by a medical research grant from the National Football League Charities Foundation and NIH grant T32 HL098069.

**Table 1:** 

Subject Characteristics and Functional Tests			
	***Linemen***	***Position Players***	***P-value***

Body Weight, kg	118.9 ± 6.8	86.9 ± 7.6	<0.0001

% Body Fat	23.6 ± 3.2	10.2 ± 2.1	<0.0001

Resting HR, bpm	63.0 ± 7.8	57.5 ± 9.5	0.2

Systolic BP, mmHg	137.6 ± 11.9	125.8 ± 9.6	0.03

Diastolic BP, mmHg	82.9 ± 8.3	79.3 ± 7.0	0.3

Hang Clean, kg	127.8 ± 12.2	117.1 ± 10.9	0.08

Back Squat, kg	164.5 ± 20.5	155.1 ± 17.7	0.3

Sumo Dead Lift, kg	179.0 ± 24.7	170.6 ± 13.7	0.4

Vertical Jump, m	0.57 ± 0.1	0.72 ± 0.1	<0.0001

Broad Jump, m	2.4 ± 0.1	2.7 ± 0.1	<0.0001

Peak Skeletal Muscle Perfusion (ml/100 g/min)			

	*Linemen *	*Position Players *	*P-value *

Soleus	153.3 ± 35.5	210.4 ± 45.7	<0.001

Gastrocnemius *	71.2 ± 13.1	128.1 ± 27.2	<0.001

Tibialis Anterior *	63.2 ± 12.7	140.7 ± 28.3	<0.0001

Fibularis Longus *	82.9 ± 20.7	134.3 ± 28.6	<0.001

*, significantly different from soleus muscle (p < 0.05).

